# Back on track – digital health applications to treat back pain of rheumatic patients? Results of a qualitative interview study

**DOI:** 10.1007/s00296-024-05726-x

**Published:** 2024-09-28

**Authors:** Katharina Boy, Susann May, Hannah Labinsky, Harriet Morf, Martin Heinze, Jan Leipe, Sebastian Kuhn, Georg Schett, Johannes Knitza, Felix Muehlensiepen

**Affiliations:** 1 Center for Health Services Research, Faculty of Health Sciences Brandenburg, Brandenburg Medical School Theodor Fontane, Rüdersdorf bei Berlin, Seebad 82/83, Berlin, 15562 Germany; 2 Department of Internal Medicine 2, Rheumatology/Clinical Immunology, University Hospital Würzburg, Würzburg, Germany; 3 Department of Internal Medicine 3, Rheumatology and Immunology Friedrich, Alexander University Erlangen-Nürnberg and Universitätsklinikum Erlangen, Erlangen, Germany; 4 Deutsches Zentrum Immuntherapie, Universitätsklinikum Erlangen, Friedrich-Alexander University (FAU) Erlangen-Nürnberg, Erlangen, Germany; 5 Department of Medicine V, Division of Rheumatology, University Medical Center and Medical Faculty Mannheim, Mannheim, Germany; 6 Institute for Digital Medicine, University Hospital of Giessen and Marburg, Philipps University Marburg, Marburg, Germany; 7 Université Grenoble Alpes, AGEIS, Grenoble, France

**Keywords:** Digital health applications, Implementation science, Rheumatology, eHealth, DTx, digital treatment, telemedicine, Non specific low back pain

## Abstract

Non-specific low back pain (NLBP) is prevalent among patients with rheumatic conditions. Digital health applications (DiGAs) provide reimbursed, personalized home treatment for patients, promising to overcome limitations of traditional healthcare systems. However, the adoption and effectiveness of back pain-specific DiGAs in rheumatology are not well understood. This study aims to explore the experiences and perspectives of a diverse group of rheumatology stakeholders regarding the use of DiGAs for back pain management. Qualitative interviews and a focus group discussion were conducted with a wide range of stakeholders including rheumatic patients, rheumatologists, nurses and DiGA producers. The data were analysed using qualitative content analysis. The study included 15 interviews (10 rheumatic patients, 4 rheumatologists, 1 DiGA producer) and 1 focus group with mixed participants (*n* = 12). Most stakeholders valued the instant access to personalized and effective back pain treatment provided by DiGAs. Patients appreciated the flexibility and ease of use of DiGAs which can be used anywhere and anytime. Concerns were raised about insufficient guidance regarding correct execution of exercises, which was seen as potentially dangerous and unsettling for patients. Healthcare professionals (HCPs) highlighted barriers, such as the lack of reimbursement, time constraints, and inadequate DiGA-specific education as barriers to prescribing DiGAs. Additionally, poor patient onboarding often led to delays, increased skepticism, and premature discontinuation of therapy. Stakeholders emphasized the challenges of current care driven by a shortage of HCPs and generally supported usage of back pain DiGAs. Various barriers and solution approaches were identified to enhance the performance, usability, and implementation of DiGAs in rheumatology.

## Introduction

Chronic non-specific low back pain (NLBP) is one of the most prevalent conditions affecting both the general population [1] and rheumatic patients [2]. NLBP can significantly impair daily activities, resulting in a reduced quality of life, an increased risk of depression, and substantial healthcare costs [1–3]. First-line treatment involves empowering patients through physical exercise, psychological interventions, and education [4]. However, in routine clinical practice, healthcare professionals (HCP) often lack the time to provide adequate and continuous support to their patients.

Digital Therapeutics (DTx) hold promise for enabling necessary patient empowerment and self-management [5]. Providing immediate access to standardized, yet personalized treatment at any time and place could help bridge the growing care gaps [6, 7]. In Germany, DTx, specifically Digitale Gesundheitsanwendungen (DiGA) [8], have been available for prescription by physicians for a three-month period and are fully reimbursed by insurance companies since October 2020. For approval, DiGAs must demonstrate safety, functionality, quality, data security, and a fundamental benefit through clinical studies. The number of available DiGAs is increasing [8], with most focusing on mental health issues, including stress and depression. Particularly in rheumatology care, which faces an increasing disease burden and a stagnating workforce, DiGAs could play a crucial role in alleviating strained healthcare services. Despite their great potential, initial studies highlight low adoption rates by rheumatologists [9]and poor patient adherence [10] of rheumatic patients. Interestingly, DTx for back pain demonstrated outstanding adherence and effectiveness rates in rheumatic patients compared to other DTx [10]. The positive results were in line with previous results from prospective large randomized controlled trials [11].

Currently, three DiGAs are available for the treatment of lower back pain in Germany: Kaia [12, 13], Vivira [11], and HelloBetter Chronische Schmerzen [14]. Kaia and Vivira primarily focus on physical exercises, while HelloBetter Chronische Schmerzen is based on cognitive-behavioral therapy. Notably, Kaia distinguishes itself from Vivira by offering smartphone camera-based exercise feedback through AI-based motion capture [15]. Whereas Kaia and Vivira lead to significant reductions of back pain, HelloBetter Chronische Schmerzen [14] did not, yet enabled a significant decrease in pain interference.

The reluctance of rheumatologists and rheumatic patients to prescribe and use back pain DiGAs, despite their promising potential, prompted this study. The aim of this study was to gain a comprehensive understanding of the experiences and perspectives of a diverse group of rheumatology stakeholders on the use of DiGAs for managing back pain. This included examining both the benefits and challenges associated with DiGAs, in order to provide a balanced view of their effectiveness, usability, and impact on patient care and outcomes.

## Methods

We conducted a qualitative study on the use of DiGAs in treatment of unspecific chronic back pain using interviews and a focus group discussion with rheumatic patients (*n* = 10), HCPs (*n* = 12) and digital health entrepreneurs (*n* = 4). The qualitative study was conducted between February and September 2023.

### Interviews & focus group

The participants were selected using purposive sampling [16] to include a heterogeneous sample in regard of age, gender, educational and professional background of the patients and HCPs and digital health entrepreneurs interviewed. We included the perspectives of rheumatic patients (*n* = 10) from the outpatient clinics of the Universitätsklinikum Erlangen that had been prescribed one of the two currently listed DiGAs to treat unspecific lower back pain (KAIA, Vivira). Additionally, we included rheumatologists (*n* = 4) that had prescribed these DiGAs before and interviewed one KAIA representative. To validate the findings of the qualitative interviews, an additional focus groups with HCPs (*n* = 8) and digital health entrepreneurs (*n* = 3) was realized.

### Data collection and analysis

The telephone interviews were conducted using an interview guide that was developed to specifically elicit the participants’ experiences. The semi-structured interview guide (Supplemental Material 1) consisted of open-ended questions that explored the user perspectives on the implementation and actual utilization of back pain DiGAs. The following main topics were investigated: the acceptance, benefits and barriers as well as the experiences with the prescription process and sustainability. The initial exploratory questions were then refined through follow-up questions. We conducted pilot interviews to test and refine the interview guide. No revisions were necessary. In addition, socio-demographic data was collected, including gender, age, diagnosis, education and occupation. We conducted a short questionnaire to systematically survey drop-outs (Supplemental Material 2). The focus group discussion was held in a video meeting. The discussion was stimulated by preliminary results of the qualitative interviews and the findings of Labinsky et al.’s study [10]. Participants did not receive financial incentives. Data based on Kuckartz’s [17] structured qualitative content analysis using MAXQDA software (Verbi GmbH). The interviews lasted between 10 and 29 (mean 15.31) minutes. After transcription of the audio material, the analysis began with a familiarization with the interviews, whereupon the interviews were coded. The categories were developed inductively to capture the relevant material in the transcripts using the data-driven development of a coding tree. Subsequently, the coding tree was applied to the entire qualitative data. At this point, the data collection had already been completed. Representative quotes from the transcripts were selected, translated into English and included in the manuscript to present the results. The manuscript has been compiled in accordance with the Consolidated Criteria for Reporting Qualitative Research (COREQ) (Supplemental Material 3) [18].

## Results

### Participant characteristics

#### Interviews

As part of this study, 23 patients that were prescribed a back pain DiGA by their rheumatologist gave their consent to telephone interviews. Eight persons could not be reached over a longer period of time (four phone calls in eight weeks) and therefore had to be excluded. Five persons stated in the preliminary telephone enquiry that they had never used or redeemed the DiGA. Ultimately we were able to conduct 10 interviews with patients that used a back pain DiGA. Mean age of interviewed patients was 38 (range: 21–58) years, see Table 1. 4/10 (40%) of patients were female. Patients reported diverse occupational and educational backgrounds. Additionally, we conducted interviews with four rheumatologists and one representative of the DiGA company KAIA. Mean age of interviewed HCP was 34 (range: 31–36) years. The entrepreneur was 39 years old. The five drop-outs who completed the short questionnaires were composed of 60% males and 40% females, with a mean age of 40 years.

#### Focus group

On September 28, 2023, we conducted a focus group with rheumatologists, rheumatology nurses, rheumatology nurse societies and digital entrepreneurs. The focus group duration was 45 min. Mean age of participants was 41 (range: 28–41) years, see Table 1.

**Table 1:** Participant characteristics

Patient	Age (years)	Gender	Diagnosis	Education	Occupation	Prescribed DiGA
**1**	57	Male	Rheumatoid arthritis	Middle School Degree	Occupational therapist	Vivira
**2**	21	Female	Suspected rheumatic disease	High School Degree	Student	Vivira
**3**	36	Male	Rheumatoid arthritis	High School Degree	Nurse	Vivira
**4**	27	Male	Rheumatoid arthritis	Middle School Degree	Master carpenter	Vivira
**5**	40	Female	Rheumatoid arthritis	Middle School Degree	Fitness specialist	Vivira
**6**	46	Female	Rheumatoid arthritis	High School Degree	Nurse	Kaia
**7**	40	Male	Rheumatoid arthritis	High School Degree	Project manager	Kaia
**8**	58	Female	Polyarthritis	Vocational training	Childminder	Kaia
**9**	25	Male	Axial spondylo-arthritis	Master’s degree	Pre-series scheduler	Kaia
**10**	31	Male	Suspected rheumatic disease	Vocational training	Cook	Kaia
**HCP**	**Age** (years)	**Gender**	**Occupation**		**Medical specialisation**	
**1**	36	Male	Consultant		Internal medicine rheumatology, physical therapy & balneology	
**2**	34	Female	Resident		Internal medicine	
**3**	33	Male	Consultant		Rheumatologist, Digital health researcher	
**4**	31	Female	Resident		Rheumatologist	
**DiGA Producer**	**Age** (years)	**Gender**	**Education**		**Occupation**	
**1**	39	Male	University degree		Kaia Health	
**Focus Group**	**Age** (years)	**Gender**	**Education**		**Occupation**	
**1**	34	Male	University degree		Research assistant	
**2**	40	Male	University degree		Physician / Specialist	
**3**	58	Male	University degree		Physician / Specialist	
**4**	61	Male	University degree		Physician / Specialist	
**5**	47	Female	Apprenticeship		Specialist assistant	
**6**	33	Male	University degree		Physician / Specialist	
**7**	36	Female	Physician /Specialist		Physician	
**8**	46	Male	University degree		Specialist assistant	
**9**	33	Male	University degree		Digital Health Entrepreneur	
**10**	28	Male	University degree		Digital Health Entrepreneur / Patient	
**11**	36	Male	University degree		Digital Health Entrepreneur	

### Themes

Four themes emerged in the qualitative content analysis: (1) Patient experiences using back pain DiGAs, (2) adoption of DiGAs by HCPs, (3) health system-related factors affecting the adoption of DiGAs, and (4) technology related DiGA improvements.

### Patient experiences using back pain DiGAs

#### Simplicity and comprehensibility

Patients found the two DiGAs to be user-friendly and easy to comprehend. One patient remarked:

“It was quite good. So it was actually quite easy to understand, everything worked well. I always found it very, very helpful when these examples were given with other people. I was also a bit relieved because I knew what they were asking for. In general, it was also very simple and understandable, in my opinion.” (P 2, pos. 31–35, Vivira).

“The instructions in it are very good, also with the demonstration, with the pictures, that’s great. Everything is also very well explained regarding times, the pauses and so on. I also like the video at the beginning, which you can watch before you start.” (P 7, pos. 5, Kaia).

#### Flexibility

The ability to use DiGAs at any time was seen as a major advantage to conventional therapy approaches:

“You have access to it 24/7, so I can do it in the evening or at night or in the morning or on the train or something. I don’t have to travel anywhere. I can also do it sometimes… So if I don’t feel like it, then I just don’t do it. I’m not tied to it in terms of time. And it’s one of those things that I can do for myself. So I don’t have to share anything with anyone, just myself and see what works for me and what doesn’t.” (P 2, pos. 39, Vivira).

#### Quality of exercises and correct posture

However, some users preferred personalized guidance to ensure the quality of exercises and correct posture, as apps cannot provide this.

“But I have to say, when you’re doing an exercise and your posture or something isn’t right, the app doesn’t notice that either. That’s why I’m someone who is really glad to have someone in front of me who can at least say once or twice, ‘Do it like this,’ or ‘You need to be careful with that,’ or ‘It’s important to me that you do it this way.” (P 4, Pos. 25–27, Vivira).

Furthermore, participants observed that the application may not fully address individual clinical presentations, particularly in the context of complex rheumatic diseases.

“And then there is this typical limitation of digitalization again. A robot cannot care for you using code. An app can accompany you on a daily basis, but doesn’t necessarily have the focus of your clinical picture right now… especially if your rheumatism is very complex. In my case, it’s primarily my hands, neck and knees. The app works in a somewhat funny rotating order and simply reaches its limits.” (P 3, pos. 21, Vivira).

### Differences between Vivira and Kaia from the user’s perspective

The Vivira and Kaia apps differ in terms of user experience. Vivira requires interaction in the form of frequent clicking during the exercises and often leads to uncertainty during exercise, which is why users emphasized the benefits of personal guidance, for example from physiotherapists. Kaia, on the other hand, appeared to offer a more seamless and user-friendly exercise sessions also offering performance feedback via motion capture.

“When you’ve used both apps, you naturally start comparing them. Overall, I wasn’t very happy with Vivira. One issue was the user interface - I think it wasn’t very well designed. d. But the main thing that bothered me was the actual usage. With the Vivira app, you had to click a lot during the exercises. You had to constantly click to continue or to confirm that you had understood the exercise before moving on to the next one, which was quite annoying. That involved a lot of clicking. So the classic scenario where you just sit or stand and go through the exercises for five or ten minutes without interruption didn’t exist, and I found that quite tiring. With Kaia, the process is smoother. The session is continuous. As soon as you press start, it runs through to the end. In other words, you can click, but you don’t have to. I find that much more convenient.” (P 8, Pos. 17–21, Kaia).

#### Personal motivation

The personal motivation and individual intensity of suffering were found to be related to DiGA usage: Patients reported that the frequency of use decreased after some time following the prescription.

“So in the beginning, well, of course, I had a quick check in the evening and picked up one or two things. And then at some point it just wore off. This impulse was simply gone.“.

(P 4, pos. 13, Vivira)

“I think I didn’t keep track of it as much because I didn’t have many more symptoms. It stabilised well and if it doesn’t hurt, then you don’t do as much.” (P 6, pos. 27, Kaia).

“Simply personal convenience – I often used it in the evening, if I used it at all. I have two young children, which means that the evening, the evening routine, is relatively long and exhausting. And it’s often when there’s actually free time… I often don’t think about using the app.” (P 8, pos. 33, Kaia).

### Adoption of DiGAs by HCP

#### Healthcare Professional perspectives

HCPs highlighted the potential of DiGAs to improve efficiency in healthcare through scalable and personalized treatment methods. They emphasized DiGAs’ potential for long-term pain reduction and prevention of acute episodes. A rheumatologist has closely described his perspective on the process of DiGA use in the interview:

“That these DiGAs are available. So beforehand, the problem has been discussed with the patient, they have been asked about their therapy requirements and whether they can handle and use them. And then you can address the DiGAs, which are new, which is basically medication, digital medication, which is also recommended and tested by the *Bundesinstitut für Arzneimittel und Medizinprodukte* [Federal Institute for Drugs and Medical Devices]. I think this is important information with the BfArM, which then also characterizes it as useful. I provide a medical prescription and briefly explain that the app can be downloaded from the App Store. I explain to the patients that they will then receive an activation code that they can use in the app to activate and use it. They should then do this for three months and then give me feedback on whether it has helped.” (Ph 1, pos. 21).

#### Need for education

However, HCPs also stressed the need for better information and education about DiGAs for both patients and physicians.

“So it took me a while to use the prescription. Well, because I was sceptical first. […] I’ve had musculoskeletal problems for a long time and have been going to the physiotherapist for ages and have always been handed a piece of paper with “you have to do these exercises.” And I always did and it just didn’t get any better. I just thought the app might be a slightly better exercise sheet to take home. I first had to understand what it was all about.” (P 10, pos. 15–19, Kaia).

“There is not enough information directly for physicians.

It is possible to look at the DiGA catalogue and read through it, of course, but I think it is very important for physicians or colleagues that the app is safe and delivers good results. I don’t think there is such a general overview. I mean, you can’t see the most important information at a glance, which might be important for your speciality. And I believe that if you have this background information as a physician, then you can use a questionnaire to see whether the patient is even suitable for it. But I think it’s more important for the physician to first know what benefits the app is supposed to bring, such as improving mobility, reducing pain, and is it safe to use. So you don’t prescribe anything that might harm the patient.” (Ph 2, pos. 39).

#### Role in DiGA implementation

HCPs had diverse views on their role in implementing DiGAs, with some supporting the process actively and considering it as rather easy and others viewing it as beyond their responsibility and too time consuming:

“I briefly explain to the patients that they have to download the app from the app store and send the prescription to the health insurance company, then receive an activation pin that activates the app. Patients should use it for three months and I ask them to give me feedback on whether it has helped.” (Ph 1, Pos. 21).

“Well, I do explain the app, tell patients how it is structured, that there is the option of doing exercises that are relatively short. I recommend that patients should make sure to do 15 min of exercises every day, as the app specialises over time. […] I always explain briefly that it is also evidence-based, i.e. that there was also a pivotal study to see whether pain improves. […] But I think we could actually say a lot more about it and perhaps do a test run to see whether patients really get along with the app. Because I think that can sometimes be a problem. (Ph 2, pos.15)

“I: Regarding the installation of the app or its functionality, do you provide any information?

Ph 3: No, that’s not my job “.

(Ph 3, pos. 23–24)

### Health system-related factors affecting the adoption of DiGAs

#### Health insurance companies as a DiGA bottleneck

Difficulties and delays in the prescription process were perceived as major barriers to DiGA-use. Patients and rheumatologists reported lacking coordination and standard operating procedures at the health insurance companies, which send the DiGA activation code to patients.

“It all took a very long time, probably two and a half months or more. That was a very long time. It all seemed to be new territory for my health insurance company.“.

(P 5, pos. 19–21, Vivira)

”Oh, definitely two and a half months, if that’s enough - that was a very long time. And that’s a shame, because you’re looking forward to it, then you’re waiting and you have to ask about the current status, I mean, it’s annoying. The insurance company could just get in touch if they don’t know what to do. At first they didn’t know what it was exactly, maybe they didn’t have enough information. But instead of putting my enquiry to one side, they could have contacted me. " (P 5, pos. 19–23, Vivira).

#### Increasing efficiency in DiGA prescription

Concurrently, the interviewed DiGA producer suggested simplifying the prescription process to make it more efficient:

“The patient has a health issue; the physician prescribes a paper prescription. This has to be sent to the health insurance company by post. The activation code for the digital application is sent by post - and so the question is, isn’t there an easier way to do this? By now, the time for the patient is kept quite short. We’re talking about a few days. But I think it would be technically possible to limit the whole process to a few hours, perhaps even a few minutes.” (H 1, pos. 27).

### Technical DiGA improvements

Various improvements were suggested for the two DiGAs to enhance usability and promote adherence.

#### Standalone is not enough

Patients requested additional communication options such as chat and video consultations to enable direct communication with a physiotherapist:

“I would actually find a consultation very interesting- in this context, it would probably also make sense to have a video consultation with a physiotherapist, for example, or you could also use some kind of chat tool.” (P 8, pos. 41–43, Kaia).

#### Flexible workout adjustments

Patients reported the desire for further personalization of the treatment programs, according to pain levels and sites of pain.

“That you can say, for example, that today the focus is on the back. That would be great, of course. So if you can then say what’s the problem today? Where is there a problem today? And how bad is the problem perhaps? That you say, okay, pain, NRS score of 8, whatever.” (P 3, pos. 21, Vivira).

#### Login process optimization

The optimization of basic functions, such as improving and simplifying the login process to minimize potential barriers, i.e. forgotten passwords, was recommended:

“I don’t know exactly what the problem was, but as soon as the app was closed I had to log in again. In other words, the access was not actively open. And that’s always really annoying because I rarely write down the password anywhere, especially for apps that I don’t use all the time. And then the password reset process starts - I’ve had to use it once or twice now - and it takes a bit longer. It always took between half an hour, sometimes even a little longer, for the email to arrive. On days like that, I didn’t use the app because I couldn’t log in.” (P 8, pos. 35, Kaia).

#### Additional features

Participants expressed a desire for additional features such as timers, reminder functions, mood barometers, motivational feedback, and pain scales:

“So I think that perhaps somehow a psychological aspect should be included, the psyche also has a major role to play, right? That would be important to me - to actively state the mood.” (P 5, pos. 48, Vivira).

*“*What would have helped me would perhaps have been something that motivates me more to achieve goals. Like you can find in some language apps, achievements in between.” (P 10, pos. 35, Kaia).

#### Expanding device compatibility

Developing a version of the app that works on desktop computers or laptops (web-app) was also suggested to give users more flexibility in their device choice:

“I’d also like it if you could do this on a PC, for example, without having to download the app. If it could be done with online access, that would be cool, it would give me even more flexibility.” (P 2, pos. 51, Vivira).

#### Transparent data flows

Ensuring robust data security and providing clear information about data handling were highlighted as essential:

“I ask myself, how is my data handled? Can my data be accessed? Could I tell you now, oh, that was marvellous, that was great, and then you say, that’s not true, you weren’t using the app at all! Will it then be passed on to my doctor? So that’s something that also concerned me, where I thought to myself what do they want to know and what will be done with it or something.” (P 1, pos. 93–95, Vivira).

### Synthesis

**Fig. 1:**
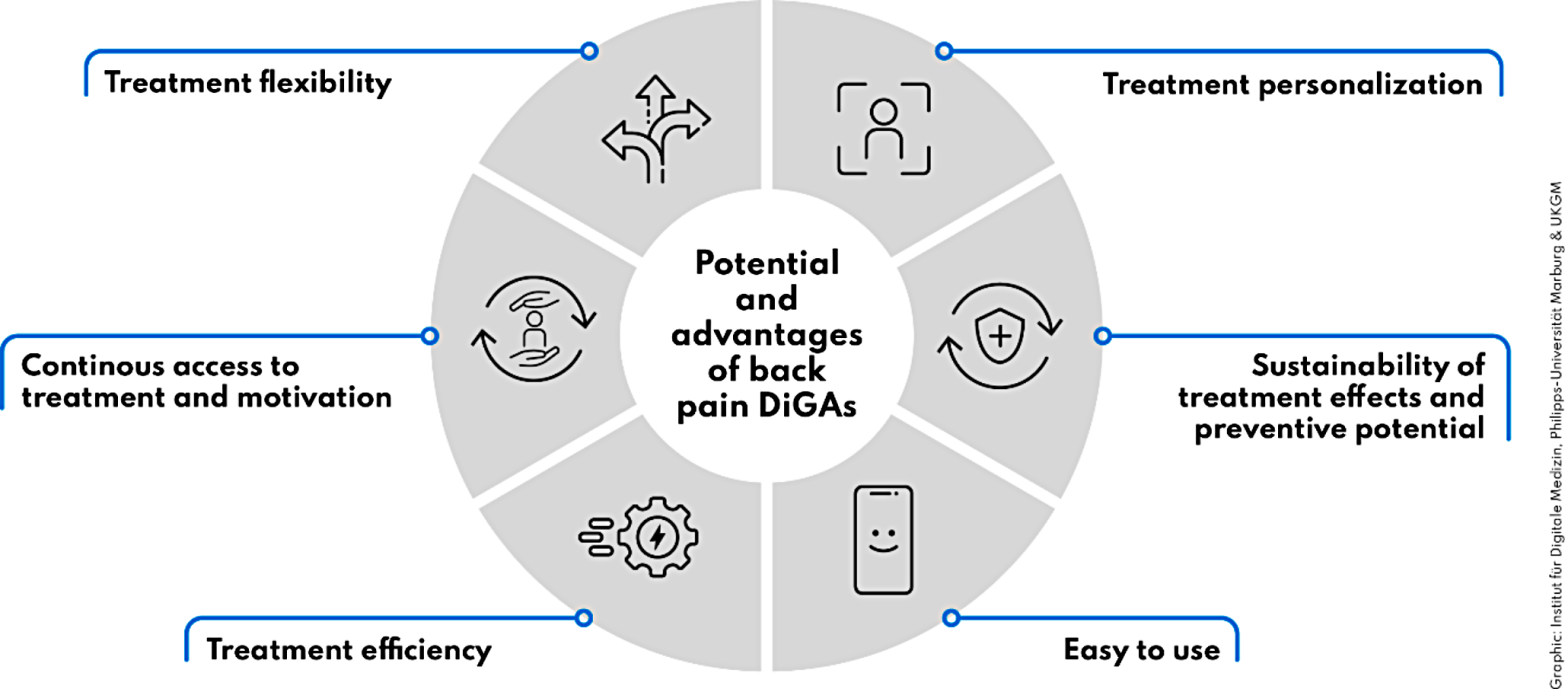
Summary of perceived advantages and strenghts of back pain DiGAs

**Fig. 2:**
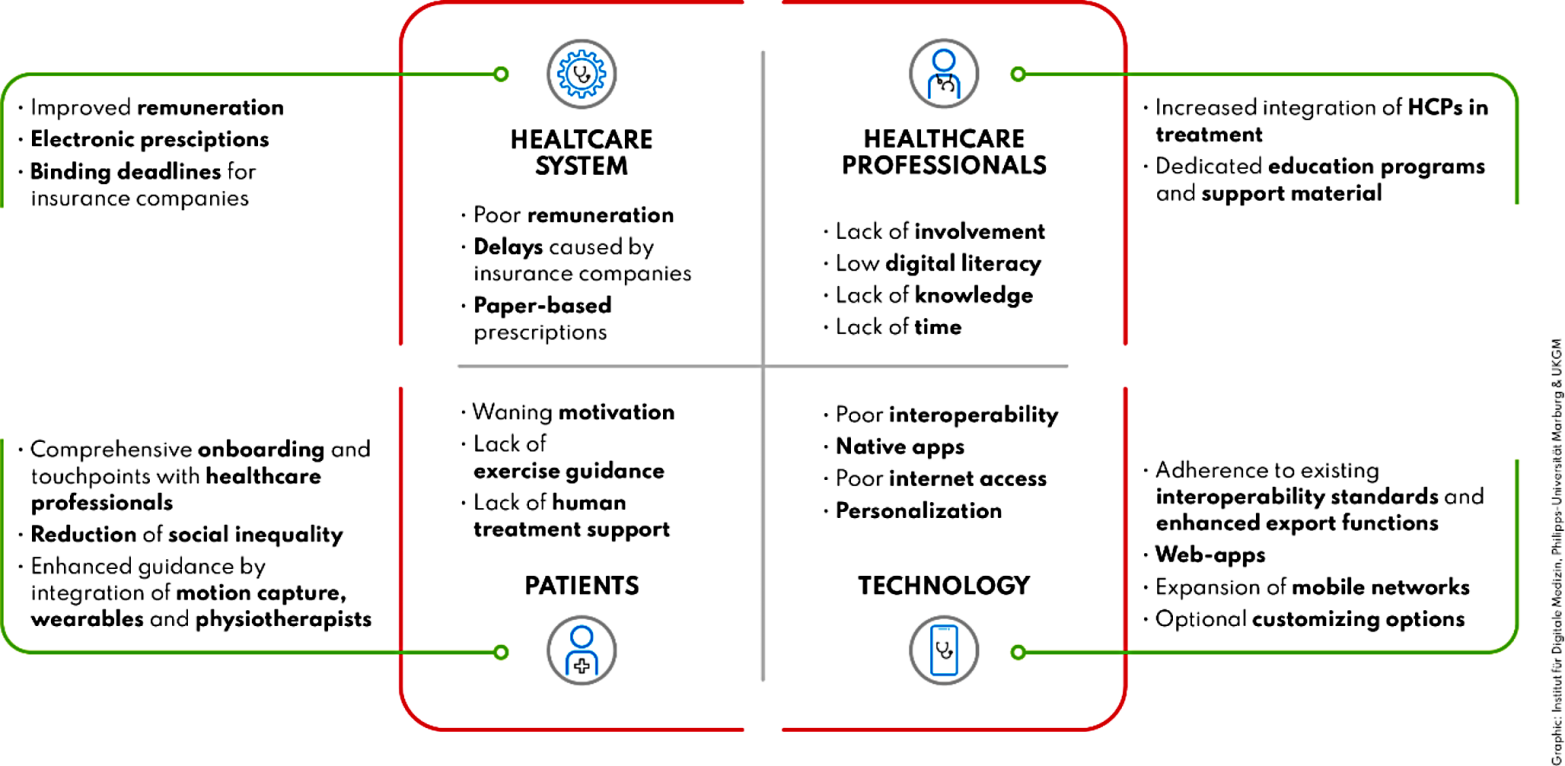
Summary of observed implementation barriers and potential approaches to overcome them, categorized into four dimensions: patients, healthcare professionals, technology, and the healthcare system

The interview participants’ reports can be synthesised into perceived back pain DiGA advantages and strengths (Fig. 1) as well as implementation barriers and potential approaches to overcome them (Fig. 2).

## Discussion

The aim of this study was to to gain a comprehensive understanding of the experiences and perspectives of a diverse group of rheumatology stakeholders on the use of DiGAs for managing back pain. This included examining both the benefits and challenges associated with DiGAs, in order to provide a balanced view of their effectiveness, usability, and impact on patient care and outcomes. To our knowledge, this is the first qualitative study evaluating DiGAs in rheumatology. Understanding the perspectives of relevant stakeholders is essential for the sustainable and effective implementation of back pain DiGAs in rheumatology care.

Patients found DiGAs user-friendly and flexible, integrating them into their daily routines with ease. However, they highlighted the limitations of DiGAs in addressing complex rheumatic conditions and expressed a need for enhanced guidance, ideally provided by physiotherapists rather than automated systems. HCPs recognized the potential of DiGAs to enhance healthcare efficiency but emphasized the need for dedicated education and information for both patients and providers. Significant barriers were identified within the healthcare system, including poor remuneration, and lengthy, uncoordinated, and paper-based prescription processes. Suggested improvements included accelerating the fully electronic prescription process with binding deadlines to deliver activation codes to patients. Fortunately, electronic prescriptions have recently been introduced into clinical practice in Germany, and an update to the law now obligates statutory insurers to provide patients with an activation code within 48 h.

Identified barriers, such as obtaining activation codes from insurance companies and challenges with DiGA adherence, align with Labinsky et al. [10], who reported that only 51% of rheumatic patients download a DiGA after prescription, and only 13% complete the entire DiGA program. Dahlhausen et al. [19] also depicted challenges in the prescription and activation process. Low DiGA adherence and considerable manufacturer prices for a period of 3 months (Vivira: €206.79, Kaia: €221.49 [8]) raise concerns among health insurance companies [12]. Implementing pay-for-performance pricing models could help reduce costs while maintaining access to treatment [20]. Identified barriers such as need for HCP education, additional HCP support (hybrid/blended treatment), and lack of HCP time for patient onboarding, are in line with recent findings from a qualitative study investigating DiGA usage among general physicians [21].

Interestingly another qualitative study in patients using apps for the treatment of low back pain reported no fear of doing anything wrong while exercising or that they would have felt safer under the supervision of a therapist [22]. In this study patients received additional messages from their physicians, which were perceived very positively. A recent randomized controlled trial [23] demonstrated significantly higher patient adherence and satisfaction, however no significant on pain reduction, when app-based therapy was augmented by biweekly face-to-face appointments, highlighting the potential benefit of a blended therapy approach, that has also been demonstrated for cognitive behavioral therapy [24]. An additional barrier specific to rheumatology is the absence of dedicated Digital Health Applications (DiGA) for inflammatory rheumatic diseases. However, increasing evidence highlights the effectiveness and high patient acceptance of medical exercise apps for patients with axial spondyloarthritis [10, 25, 26] who suffer from inflammatory back pain. Interestingly, while some patients distinctly prefer face-to-face therapy, others value the convenience and benefits of app-guided exercises at home [25].

Consequently, we have formulated recommendations that could facilitate the sustainable integration of DiGAs into rheumatology care and the healthcare system in general.

Based on the findings of our study, we propose several recommendations aimed at facilitating the sustainable integration of DiGAs into rheumatology care and the healthcare system in general. These recommendations address the needs and perspectives of healthcare professionals (HCPs), patients, the healthcare system, and technology enhancements.

HCPs require comprehensive information and continuous training on the benefits, usage, and potential limitations of DiGAs. This will enable them to make informed decisions and effectively prescribe and support the use of DiGAs in clinical practice. Providing patients with detailed information and training on how to use DiGAs effectively can enhance adherence and improve outcomes. This includes educating patients about the potential benefits, usage instructions, and addressing any concerns or misconceptions they may have.

Implementing tools and strategies to assess patients’ likelihood of adhering to DiGAs can help tailor interventions and provide additional support where needed. Identifying patients who may require more intensive follow-up can improve adherence rates. Establishing mechanisms for regular feedback and follow-up with patients after prescribing DiGAs can ensure ongoing support and address any issues that may arise during the course of their use.

Simplifying the prescription process for DiGAs is crucial for the success of DiGA implementation into German health care system. This includes reducing bureaucratic hurdles, ensuring timely delivery of activation codes, and improving coordination between HCP and insurance companies.

Enhancing the ability of DiGAs to be customized to meet individual patient needs and goals can improve user satisfaction and adherence. Personalized programs that address specific symptoms and preferences can enhance the effectiveness of DiGAs. Developing features that allow for direct communication between patients and HCPs within the DiGA platform can provide timely support and guidance. However, it is important to consider the capacity of HCPs to manage this additional communication channel.

Overall, DiGAs hold significant potential to enhance healthcare and patient engagement in rheumatology care. Addressing the identified barriers through these recommendations can promote the sustainable implementation and acceptance of DiGAs. Further research is needed to confirm these findings and explore the feasibility of the suggested improvements to increase user adherence and overall effectiveness.

To our knowledge this is the first qualitative study investigating DiGAs in rheumatology. A major strength represents the included stakeholders, including patients, rheumatologists as well as digital health company and medical society representatives. The systematic identification of barriers and respective solutions approaches (Fig. 2) is a strength, enabling practical guidance to overcome DiGA implementation barriers. This study has several limitations. As an exploratory study in the field of rheumatology with a limited sample size, more extensive research is needed to generalize the findings. No patients could be included that had received a prescription for the third approved DiGA for back pain. Inclusion of physiotherapists as a relevant stakeholder group could have augmented this study. Additionally, the perspective of insurance companies, which is crucial for understanding broader implementation challenges, was not included.

## Conclusion

Stakeholders highlighted the challenges of current care and recognized the potential of back pain DiGAs to enhance healthcare efficiency and patient self-management in rheumatology. Barriers such as delayed prescription processes, the need for more exercise guidance, and inadequate education for patients and HCPs currently limit DiGA prescription and adherence rates. Our findings provide guidance to systematically address implementation challenges in rheumatology. Further research is needed to validate these findings and explore the feasibility of the proposed implementation approaches.

## Data Availability

The datasets used and/or analysed during the current study are available from the corresponding author on reasonable request. All data relevant to the study are included in the article or uploaded as supplementary material. For further questions regarding the reuse of data, please contact the corresponding authors.

## Declarations

### Related congress abstract publication

Muehlensiepen, F., May, S., Boy, K., Heinze, M., Schett, G., & Knitza, J. (2023). [DiGAs for the treatment of comorbidities in rheumatological care: A qualitative study of patients’ perspectives.] Meeting abstract presented at the 22nd German Congress for Health Services Research (DKVF), Berlin, Germany, October 4–6, 2023. Düsseldorf: German Medical Science GMS Publishing House. 10.3205/23dkvf331.
