# Correction: Back on track– digital health applications to treat back pain of rheumatic patients? Results of a qualitative interview study

**DOI:** 10.1007/s00296-024-05747-6

**Published:** 2024-11-02

**Authors:** Katharina Boy, Susann May, Hannah Labinsky, Harriet Morf, Martin Heinze, Jan Leipe, Sebastian Kuhn, Georg Schett, Johannes Knitza, Felix Muehlensiepen

**Affiliations:** 1 Center for Health Services Research, Faculty of Health Sciences Brandenburg, Brandenburg Medical School Theodor Fontane, Rüdersdorf bei Berlin, Seebad 82/83, 15562 Berlin, Germany; 2 Department of Internal Medicine 2, Rheumatology/Clinical Immunology, University Hospital Würzburg, Würzburg, Germany; 3 Department of Internal Medicine 3, Rheumatology and Immunology Friedrich, Alexander University Erlangen-Nürnberg and Universitätsklinikum Erlangen, Erlangen, Germany; 4 Deutsches Zentrum Immuntherapie, Universitätsklinikum Erlangen, Friedrich-Alexander University (FAU) Erlangen-Nürnberg, Erlangen, Germany; 5 Department of Medicine V, Division of Rheumatology, University Medical Center and Medical Faculty Mannheim, Mannheim, Germany; 6 Institute for Digital Medicine, University Hospital of Giessen and Marburg, Philipps University Marburg, Marburg, Germany; 7 Université Grenoble Alpes, AGEIS, Grenoble, France

**Correction to: Rheumatology International**

10.1007/s00296-024-05726-x

In this article Figs. 1 and 2 were wrongly numbered; Fig. 1 should have been Fig. 2 and vice versa as shown below.

**Fig. 1:**
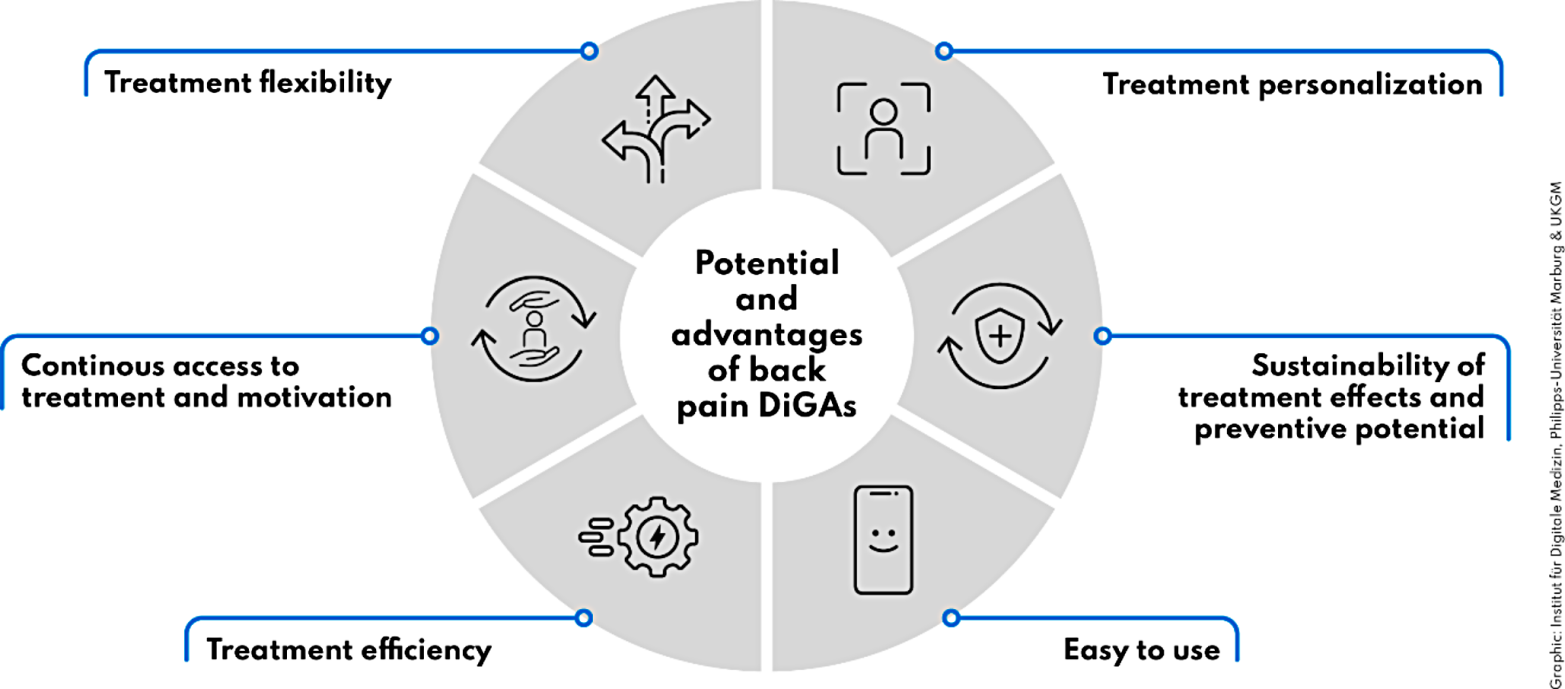
Summary of perceived advantages and strenghts of back pain DiGAs

**Fig. 2:**
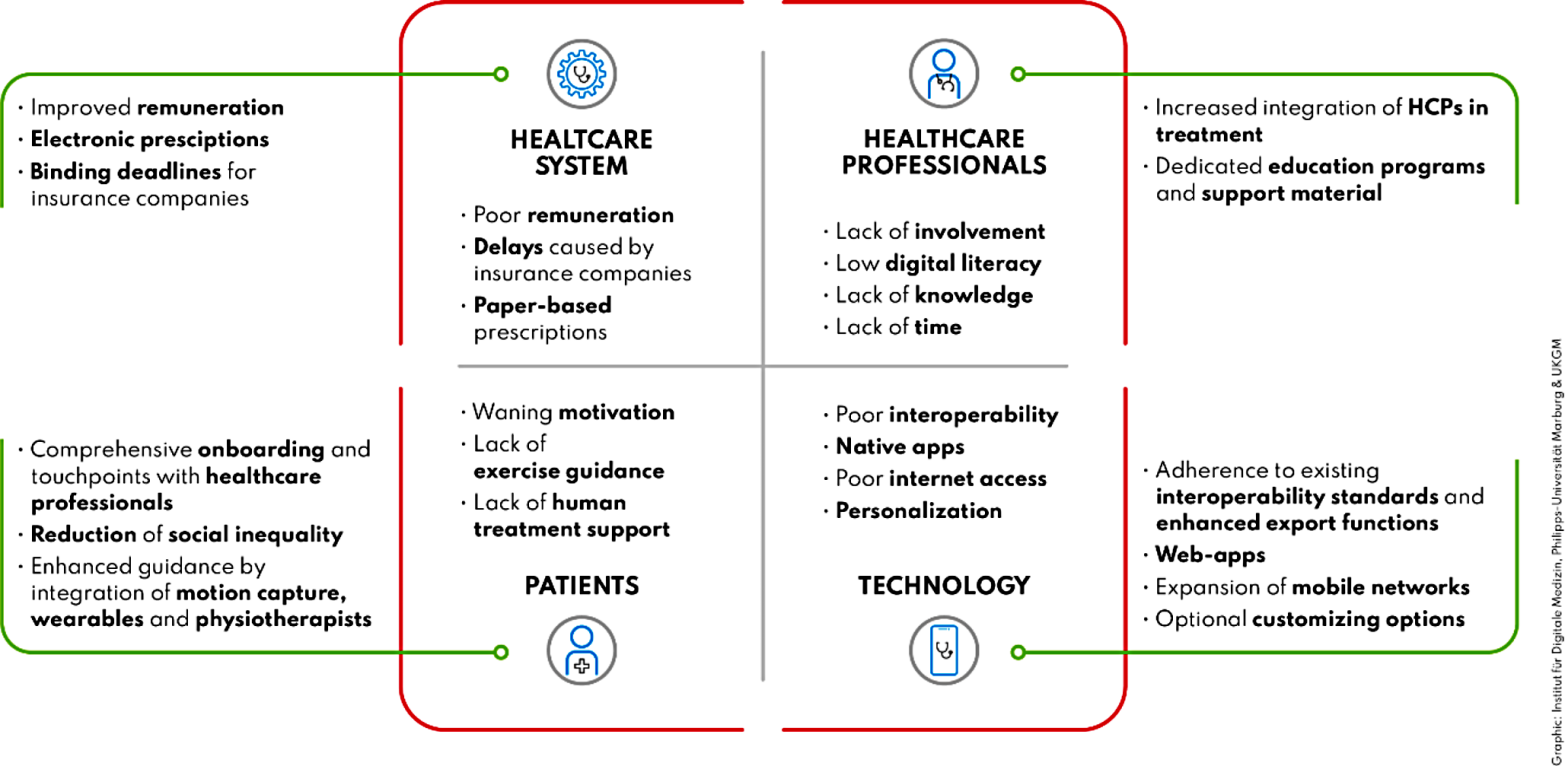
Summary of observed implementation barriers and potential approaches to overcome them, categorized into four dimensions: patients, healthcare professionals, technology, and the healthcare system

The original article has been corrected.

